# The effects of increased fluid viscosity on swallowing sounds in healthy adults

**DOI:** 10.1186/1475-925X-12-90

**Published:** 2013-09-10

**Authors:** Iva Jestrović, Joshua M Dudik, Bo Luan, James L Coyle, Ervin Sejdić

**Affiliations:** 1 Department of Electrical and Computer Engineering, Swanson School of Engineering, University of Pittsburgh, Pittsburgh, PA USA; 2 Department of Communication Science and Disorders, School of Health and Rehabilitation Sciences, University of Pittsburgh, Pittsburgh, PA USA

**Keywords:** Swallowing, Swallowing sounds, Viscosity, Signal characteristics

## Abstract

**Background:**

Cervical auscultation (CA) is an affordable, non-invasive technique used to observe sounds occurring during swallowing. CA involves swallowing characterization via stethoscopes or microphones, while accelerometers can detect other vibratory signals. While the effects of fluid viscosity on swallowing accelerometry signals is well understood, there are still open questions about these effects on swallowing sounds. Therefore, this study investigated the influence of fluids with increasing thickness on swallowing sound characteristics.

**Method:**

We collected swallowing sounds and swallowing accelerometry signals from 56 healthy participants. Each participant completed five water swallows, five swallows of nectar-thick apple juice, and five swallows of honey-thick apple juice. These swallows were completed in neutral head and chin-tuck head positions. After pre-processing of collected signals, a number of features in time, frequency and time-frequency domains were extracted.

**Results:**

Our numerical analysis demonstrated that significant influence of viscosity was found in most of the features. In general, features extracted from swallows in the neutral head position were affected more than swallows from the chin-tuck position. Furthermore, most of the differences were found between water and fluids with higher viscosity. Almost no significant differences were found between swallows involving nectar-thick and honey-thick apple juices. Our results also showed that thicker fluids had higher acoustic regularity and predictability as demonstrated by the information-theoretic features, and a lower frequency content as demonstrated by features in the frequency domain.

**Conclusions:**

According to these results, we can conclude that viscosity of fluids should be considered in future investigations involving swallowing sounds.

## Introduction

Dysphagia is a swallowing disorder [1] typically occurring in patients who suffer from a variety of neurological conditions (stroke [2], cerebral palsy [3], Parkinson’s and other neurodegenerative diseases [4]), head and neck cancer and its treatment [5], iatrogenic conditions or trauma [6] and other diseases. Dysphagia can also occur due to genetic predispositions or congenital craniofacial syndromes [7]. Among the signs and symptoms of dysphagia include the subjective sensation of difficulty swallowing food or liquids, choking or coughing before, during or after swallowing, or other symptoms caused by impaired clearance of swallowed material into the digestive system, which can cause malnutrition [8], dehydration [9], failure of the immune system [10], psycho-social degradation [11,12] and in general, a decreased quality of life [13]. A major consequence of dysphagia is aspiration of food and liquids into the airway past the vocal folds and into the respiratory system which leads to airway obstruction, pneumonia, and increased risk of mortality resulting from both [14,15].

There are several techniques for diagnosing dysphagia using imaging instrumentation. The videofluoroscopic swallowing study (VFSS) and the fiber-optic endoscopic evaluation of swallowing are the currently accepted imaging gold standards [1,16]. These diagnostic methods are available in acute care hospitals, and some rehabilitation centers and outpatient clinics, but in some settings such as nursing homes or skilled nursing facilities, they are not always readily available and patients need to be scheduled to receive these tests at a later date in the acute care hospitals. Furthermore, in some settings, immediate performance of imaging studies necessary to definitively diagnose structural or physiological swallowing disorders leading to increased risk of adverse medical events is not possible at a moment’s notice, leaving clinicians to use screening methods in an attempt to predict likely impairments and manage them while awaiting imaging assessments, despite the low precision of screening tests in the identification of impairments. In the screening of stroke patients at the acute care setting, a widely accepted standard practice in the US, immediate swallowing screening is performed upon immediately admission before the patient has had an opportunity to eat or drink or take oral medications, to identify likelihood of aspiration because aspiration and its adverse outcomes significantly increases morbidity after stroke [17]. Though imaging procedures carry some degree of invasiveness such as exposure to radiation and intubation by a fiber-optic endoscope, they remain necessary for accurate identification of impairments of swallowing function and determination of treatment options that might alleviate impairments or lower the risks caused by the impairments. They must also be performed by a trained diagnostic specialist. Therefore interest in less invasive screening methods has gained momentum over the past 20 years. A non-invasive method of screening for dysphagia known as cervical auscultation (CA) has been explored in recent years [18], although its ability to identify or predict specific features of dysphagia or guide intervention to alleviate risks associated with dysphagia has not been established [19]. CA usually involves investigating signals acquired via stethoscopes or microphones [20,21]. As in all noninvasive screening methods, one attraction of CA is its mobility for day-to-day monitoring and a low price [21] though its predictive value for identifying important diagnostic signs has yet to be established. CA as a tool for screening for dysphagia is still under investigation (e.g., [18,21]).

Previous studies indicated that thicker liquids can reduce the amount of material that is aspirated when individuals aspirate thin liquids while swallowing [22] or subjectively improve swallowing symptoms in some individuals who have dysphagia with ordinary liquids so it would be informative to determine whether the effects of increased fluid viscosity on swallowing signal characteristics produces useful information that might add value to auscultation as a screening method [23,24]. Though there is understanding of the effects of increased viscosity on swallowing accelerometry signals (e.g., [25]), the effects on swallowing acoustics are more challenging to understand. One challenge is that previous studies used microphones of a varying quality to acquire swallowing sounds. In [26], the authors used Sony ECM-C115 microphone with a frequency response from 50 Hz to 15 kHz to show that duration of the swallow signals are longer for thicker fluids. A similar trend was observed by Reynolds *et al.*[27] using an electret microphone Optimus (Radio-Shack/Tandy Corp, Model 333013), with a nonlinear frequency response form 70 Hz to 16 kHz. Other challenges to the usefulness of auscultation in dysphagia screening stems from the previously adopted microphones, which were not able to capture low frequency components of swallowing sounds. In our recent study Dudik JM, Jestrović I, Luan B, Coyle J, Sejdić E: A comparative analysis of swallowing accelerometry and sounds during saliva swallows. [Submitted], we showed that the swallowing sounds are centered at lower frequencies below 50 Hz and their bandwidth extends up to few hundred Hertz. These open challenges prompted us to conduct the current investigation.

In this paper, we sought to investigate the effects of fluids with increased viscosity on swallowing sound characteristics. In particular, we examine the signal characteristics in time, frequency and time-frequency domains, while participants completed swallows in neutral head-neck posture and the head-neck flexion (chin-tuck) position which has also been used to manage aspiration in patients with specific biomechanical swallowing impairments [28-31]. To compare our results with the previous study [25], we also simultaneously collected dual-axis swallowing accelerometry signals.

## Methodology

### Data acquisition from participants

In this study, simultaneous accelerometry and acoustic data were collected from 56 healthy adults aged 18 to 65 years. All subjects had no previous history of neurological diseases, swallowing difficulties and/or cancer of the mouth, neck or brain. The study protocol was approved by Institutional Review Board at the University of Pittsburgh.

**Figure 1:**
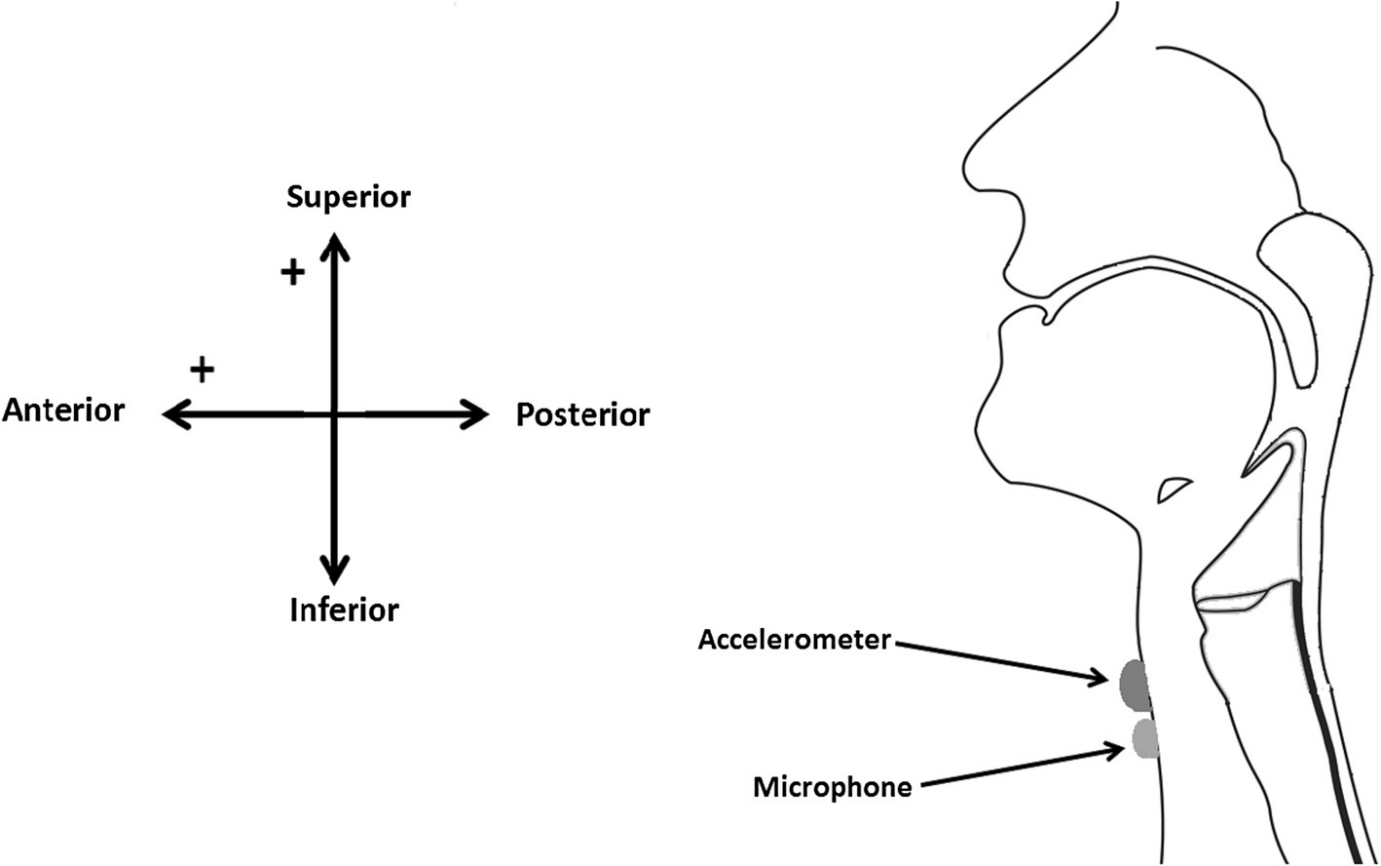
Position of accelerometer and microphone.

After signing a consent form and recording information about subjects’ height and weight, the dual-axis accelerometer (ADXL322, Analog Devices, Norwood, MA, USA) and contact microphone (AKG C411L, AKG Acoustics GmbH, Vienna, Austria) were attached to the subject’s neck with double sided tape. The accelerometer was placed below the thyroid cartilage and the microphone was placed below the accelerometer, far enough to avoid contact between two sensors, as shown in Figure 1. The experimental procedure was divided in to two parts conducted in the same order for all participants. First, participants completed bolus swallows in a neutral-head position, followed by the completion of swallows in a chin-tuck position. In both parts, the subject was asked to take five individual swallows of different fluids: water, nectar consistency and honey consistency apple juices. Thickened apple juices are commercially available products (Nestlé Health Care, Inc. Florham Park, NJ, USA). Nectar consistency and honey consistency apple juices are classified by the Australian Standard for Texture Modified Foods and Fluids, as Mildly Thick-Level 150 for nectar and Moderately Thick-Level 400 for honey-thick. All fluids were served chilled (3-5°C) in cups as approximately one bolus per cup. Participants were asked to complete the individual swallows of a single bolus at a comfortable pace while consuming comfortable volumes of fluids. The volume of bolus was not controlled as there are sex based differences in a comfortable bolus size [32]. We intend to investigate the effects of specific bolus volumes in future research.

The accelerometer was powered with a 3V output power supply (1504 DC/AC Power Supply, B&K Precision Corporation, Yorba Linda, CA, USA). The two axes of the accelerometer were positioned in the anterior-posterior (A-P) and superior-inferior (S-I) directions. Signals from both axes of the accelerometer were passed through an amplifier (P55, Grass Technologies, Warwick, RI, USA), which provided 10 times amplification and then were band-pass filtered from 0.1 to 3000 Hz. A microphone was powered by a power supply (model B29L, AKG, Vienna, Austria). Swallowing accelerometry signals and swallowing sounds were sampled at 40 kHz by the LabView program Signal Express (National Instruments, Austin, TX, USA) running on a personal computer. All data were saved on an external hard drive.

### Pre-processing steps

First, all collected signals were pre-processed according to previously proposed algorithms (e.g., [25]). The accelerometer signals were downsampled to 10 kHz in order to implement previously proposed approaches [25].

All acquired signals were initially filtered with a finite impulse response (FIR) filter to annul the effects of the data acquisition equipment. The filters for swallowing accelerometry signals and swallowing sounds were designed according to the procedure outlined in [33] using 18 table-top recordings in a quiet room.

Next, we removed very low frequency components from the dual-axis accelerometry signals associated with head movements [34]. Since the microphone signal was not affected by any head movements, there was no need to perform such an operation for these signals.

Consequently, all signals were denoised with 10-level discrete wavelet decomposition using the discrete Meyer wavelet with soft-thresholding using the global denoising threshold, *T*_*den*_ defined as:

(1) $$T_{\text{den}}=\frac{\text{med}(|d_{1}|)\sqrt{2\log n}}{0.6745},$$

where *d*_1_ represents wavelet coefficients at the first level, *n* is length of the signal and *med* is median operator [35].

The last pre-processing step was the segmentation of signals carried out according to the sequential fuzzy c-means algorithm designed for dual-axis accelerometry signals [36]. All segmentation results were verified visually, if any of them were incorrect, swallows were segmented manually. Swallows which could not be segmented were excluded from the study (less than 5% were excluded). The time instances identified in this process as the beginning and the end of each swallow were then used to segment the microphone signals.

After the completion of all pre-processing steps, the features outlined in the next subsection were extracted from each swallow.

### Feature extraction

Each swallowing sound could be represented as a discrete time series, *M*={*m*_1_,*m*_2_,…,*m*_*n*_}. Different signal features can be used to describe swallowing characteristics, and we summarize below the features considered in this study. The same set of features was considered for both swallowing sounds and dual-axis swallowing accelerometry signals.

#### Time domain features

•The mean (average) value of a signal represents unbiased estimation of the amplitude of the signal. An equation for calculating the mean value is given as

(2) $$\mu_{m}=\frac{1}{n}\overset{n}{\underset{i=1}{\sum }}m_{i}.$$

•The standard deviation is a measure of variation from the mean value. It can be obtained as

(3) $$\sigma =\sqrt{\frac{1}{n-1}\overset{n}{\underset{i=1}{\sum }}{\left(m_{i}-\mu_{m}\right)}^{2}}.$$

•The skewness represents symmetry of a distribution of the signal [25]. It can be calculated as,

(4) $$\nu =\frac{\frac{1}{n}\overset{n}{\underset{i=1}{\sum }}{\left(m_{i}-\mu_{m}\right)}^{3}}{{\left(\frac{1}{n}\overset{n}{\underset{i=1}{\sum }}{\left(m_{i}-\mu_{m}\right)}^{2}\right)}^{1.5}}.$$

•The kurtosis is a measure of the “peakedness” of the probability distribution of a variable. For a high value of kurtosis, the distribution is sharp and narrow, with heavy tails. A low kurtosis value indicated a flat distribution peak and thin tails. Kurtosis is calculated as

(5) $$\varpi =\frac{\frac{1}{n}\overset{n}{\underset{i=1}{\sum }}{\left(m_{i}-\mu_{m}\right)}^{3}}{{\left(\frac{1}{n}\overset{n}{\underset{i=1}{\sum }}{\left(m_{i}-\mu_{m}\right)}^{2}\right)}^{2}}.$$

•The entropy rate [37,38] quantifies the extent of regularity in a signal. It provides important information about swallows as an random process. Entropy rate is calculated in several steps. First, a signal *M* should be normalized to zero mean and unit variance. The normalized *M* is then quantized to 10 equally spaced levels. Those 10 levels are ranged from minimum to maximum and marked with integer numbers from 0 to 9. Then the quantized signal $\hat{M}=\{{\hat{m}}_{1},{\hat{m}}_{2},\ldots ,{\hat{m}}_{n}\}$, with *U* consecutive points is coded as

(6) $$s_{i}={\hat{m}}_{i+U-1}\cdot 10^{U-1}+\ldots +{\hat{m}}_{i}\cdot 10^{0},$$

•where *i*=1,2,…,*n*−*U*+1, and *S*_*i*_={*s*_1_,*s*_2_,…*s*_*n*−*U*+1_} are coded integers. Because of the 10 quantization levels, 10 is used as a base. Using the Shannon entropy formula, the entropy is estimated as

(7) $$E(U)=-\overset{10^{U-1}}{\underset{k=1}{\sum }}P_{S_{u}}(k)\cdot \text{ln}P_{S_{U}}(k),$$

•where $P_{S_{u}}$ is probability of observing *k* in *S*_*u*_, approximated by the corresponding sample frequency. The entropy is then normalized using following formula

(8) $$\hat{\text{NE}(U)}=\frac{E(U)-E(U-1)+E(1)\cdot \alpha }{E(1)},$$

•where *α* is the percentage of the coded integers in *S*_*i*_ that occurred only once. Finally, the regulatory index as a measure of the entropy rate is calculated as

(9) $$\rho =1-\text{min}\hat{\text{NE}(U)}.$$

•*ρ* takes value from 0 to 1, where for regulatory index is equal to 1 indicates maximum of regularity, while value of 0 represents maximum of randomness.

•The Lempel-Ziv complexity (L-Z) [39] provides information about predictability of the signal. To compute the L-Z complexity, a signal *M* should be first quantized into 100 equally spaced levels. Then this 100 levels are ranged from minimum to maximum values. In the next step, the quantized signal $A_{1}^{n}=\{a_{1},a_{2},\ldots ,a_{n}\}$ was decomposed in *L* different blocks of the length *l*−*j*+1, so that $A_{1}^{n}=\{\psi_{1},\psi_{2},\ldots ,\psi_{n}\}$. Blocks are defined as

(10) $$\Psi =A_{1}^{n}=\{a_{j},a_{j+1},\ldots ,a_{l}\},1\leq j\leq l\leq n$$

•The first block is equal to the first element of the quantized signal. Other blocks are defined as

(11) $$\Psi_{m+1}=A_{h_{m}+1}^{h_{m+1}},m\geq 1,m\in {\mathbb{Z}}^{+}$$

•where *h*_*m*_ is ending index for *ψ*_*m*_. Finally, the L-Z complexity is calculated as

(12) $$\text{LZ}=\frac{L\log_{100}n}{n}$$

#### Frequency domain features

•The peak frequency of a signal is defined as

(13) $$f_{p}=\underset{f\in [0,f_{\text{max}}]}{\text{argmax}}|F_{M}(f){|}^{2},$$

•where *f*_*max*_ is the highest available frequency in a signal and *F*_*M*_ represents the Fourier transform of a signal.

•The centroid frequency indicates position of the center of mass in the signal in the frequency domain [33]. For the signal, *M*, it is estimated as

(14) $$f_{c}=\frac{\overset{f_{\text{max}}}{\underset{0}{\int }}f|F_{M}(f){|}^{2}\text{df}}{\overset{f_{\text{max}}}{\underset{0}{\int }}|F_{M}(f){|}^{2}\text{df}}.$$

•Bandwidth represents spectral spread and it is defined as

(15) $$\text{BW}=\sqrt{\frac{\overset{f_{\text{max}}}{\underset{0}{\int }}{\left(\;f-f_{c}\right)}^{2}|F_{M}(f){|}^{2}\text{df}}{\overset{f_{\text{max}}}{\underset{0}{\int }}|F_{M}(f){|}^{2}\text{df}}}.$$

#### Time frequency domain feature

•The relative energy was computed using a 10-level discrete wavelet decomposition of the signal with the Meyer wavelet [25,40-42]. The energy at each decomposition level is computed using the Euclidean norm of decomposition coefficient vectors:

(16) $$E_{a_{10}}=||a_{10}|{|}^{2},$$

 

(17) $$E_{d_{i}}=||d_{i}|{|}^{2},$$

•where *a*_10_ is the approximation signal and *d*_*i*_ is detail signal. The total energy was calculated as

(18) $$E_{T}=E_{a_{10}}+\overset{10}{\underset{i=1}{\sum }}E_{d_{i}},$$

•Finally, percent of relative energy contribution from each decomposition level was computed as

(19) $$E_{t_{a_{10}}}=\frac{E_{a_{10}}}{E_{T}}\times 100\%,$$

 

(20) $$E_{t_{d_{i}}}=\frac{E_{d_{i}}}{E_{T}}\times 100\%,$$

for *i*=1,2,…,10.

•Wavelet entropy describes the information distribution in the time-frequency domain. Wavelet entropy was computed using 10-level wavelet decomposition and relative energy computed above, with following formula:

(21) $$\text{WE}=-\frac{E_{t_{a_{10}}}}{100}\cdot {\text{log}}_{2}\frac{E_{t_{a_{10}}}}{100}-\overset{10}{\underset{i=1}{\sum }}\frac{E_{t_{d_{i}}}}{100}\cdot {\text{log}}_{2}\frac{E_{t_{d_{i}}}}{100},$$

### Data analysis

The statistical differences between different conditions were tested using a non-parametric statistical hypothesis test, Wilcoxon rank-sum test [43].

## Results

Results of the feature extraction process are presented as a mean value ± standard deviation. We analyzed 271 water swallows in neutral position and 274 in chin-tuck position, 277 nectar-thick apple juice in neutral position and 275 in chin-tuck position, and 273 honey-thick apple juice swallows in neutral position and 273 in the chin tuck position.

### Time domain features results

Table 1 summarizes the time domain features from the swallowing sounds. The results showed that standard deviation (*σ*), skewness (*ν*) and kurtosis (*ϖ*) were not significantly different between the control condition (water) and the thickened liquid conditions in the chin-tuck position (*p*>0.05, *zval*<−0.49, *ranksum*<76218). For the swallows in the neutral position, pairwise comparison between water and nectar-thick apple juice revealed statistically significant differences for standard deviation (*p*=0.03, *zval*=2.23, *ranksum*=81398) and skewness (*p*=0.01, *zval*=−2.53, *ranksum*=72315). The skewness was significantly different between water and honey-thick apple juice (*p*<<0.01, *zval*=−3.73, *ranksum*=69545) as well as the kurtosis (*p*=0.02, *zval*=2.51, *ranksum*=765150). Next, we observed significantly higher entropy rates (*ρ*) for nectar-thick and honey-thick fluids in comparison to water for both head positions (*p*<<0.01, *zval*<−4.09, *ranksum*<70304). However, the L-Z complexity had statistically the highest values for water swallows for both head maneuvers (*p*<0.05, *zval*<3.22, *ranksum*<805970).

**Table 1:** Time domain features for swallowing sounds

		**Neutral position**			**Chin-tuck position**	
**Feature**	**Water**	**Nectar-thick**	**Honey-thick**	**Water**	**Nectar-thick**	**Honey-thick**
		**apple juice**	**apple juice**		**apple juice**	**apple juice**
*σ*	0.54±0.03	0.42±0.02	0.54±0.03	0.54±0.02	0.54±0.02	0.54±0.02
*ν*	−1.34±0.22	−0.80±0.20	−1.04±0.34	−1.53±0.41	−2.19±0.59	−0.69±0.43
*ϖ*	92.5±17.1	96.1±16.7	173±43.1	157±37.5	300±57.7	227±41.6
*ρ*^∗^	98.7±0.04	99.0±0.04	99.1±0.06	98.1±0.14	98.5±0.10	98.7±0.05
*LZ*^∗^	6.14±0.15	5.78±0.16	5.61±0.18	7.45±0.29	6.39±0.26	5.98±0.20

* denotes multiplication by 10^-2^.

Table 2 summarizes the results for the swallowing accelerometry signals. The results showed that in the A-P direction of the accelerometer signal, standard deviation and kurtosis in the chin-tuck position were not affected by the fluid viscosity (*p*>0.05, *zval*<2.69, *ranksum*<82959). Water swallows in the neutral position had the statistically highest values for standard deviation (*p*<0.01,*zval*<3.67,*ranksum*<83135) and the lowest values for kurtosis (*p*<0.03,*zval*<3.95,*ranksum*<84631). The skewness was statistically different between nectar-thick and honey-thick apple juice in neutral position (*p*=0.03,*zval*=−2.01,*ranksum*=74794), and between water and honey-thick apple juice in chin-tuck position (*p*=0.03,*zval*=−2.48,*ranksum*=73126). Furthermore, water swallows had statistically the lowest values for entropy rate (*p*<0.05,*zval*<−5.01,*ranksum*<68555) and the highest values for the L-Z complexity (*p*<<0.01,*zval*<6012,*ranksum*<834210) in comparison to other two fluids in both head positions. Also, a pairwise comparison between nectar-thick and honey-thick swallows found significant differences for entropy rate (*p*=0.01,*zval*=−2.03,*ranksum*=74383) and L-Z complexity (*p*=0.03,*zval*=−2.87,*ranksum*=83728) in the head chin-tuck position.

**Table 2:** Time domain features for swallowing accelerometry signals

		**Neutral position**			**Chin-tuck position**	
**Feature**	**Water**	**Nectar-thick**	**Honey-thick**	**Water**	**Nectar-thick**	**Honey-thick**
		**apple juice**	**apple juice**		**apple juice**	**apple juice**
*σ*^∗^ A-P	1.39 ±0.05	1.16 ±0.03	0.39 ±0.02	1.39 ±0.04	1.39 ±0.04	1.39 ±0.04
*σ*^∗^ S-I	1.11 ±0.06	0.96 ±0.03	1.16 ±0.05	1.16 ±0.05	1.16 ±0.04	1.16 ±0.05
*ν* A-P	-0.73 ±0.22	-1.39 ±0.23	-0.74 ±0.21	-2.31 ±0.43	-2.24 ±0.49	-1.31 ±0.42
*ν* S-I	0.28 ±0.32	0.14 ±0.37	-0.49 ±0.39	-0.13 ±0.31	-0.69 ±0.29	-0.54 ±0.37
*ϖ* A-P	64.5 ±12.8	62.7 ±16.7	64.1 ±13.6	173 ±30.5	193 ±42.1	183 ±33.6
*ϖ* S-I	81.8 ±17.0	121 ±28.1	118 ±32.2	96.9 ±21.2	193 ±21.5	145 ±22.6
*ρ*^∗^ A-P	98.8 ±0.04	99.1 ±0.02	99.1 ±0.04	98.5 ±0.07	98.8 ±0.06	99.1 ±0.04
*ρ*^∗^ S-I	99.1 ±0.03	99.2 ±0.02	99.2 ±0.03	98.5 ±0.08	98.8 ±0.04	98.9 ±0.04
*LZ*^∗^ A-P	5.46 ±0.12	4.97 ±0.12	4.92 ±0.14	6.26 ±0.19	5.44 ±0.17	4.83 ±0.14
*LZ*^∗^ S-I	6.36 ±0.14	6.21 ±0.15	6.31 ±0.16	7.17 ±0.22	6.42 ±0.21	5.91 ±0.18

* denotes multiplication by 10^-2^.

In the S-I direction, the fluid thickness did not have influence on L-Z complexity in the head neutral, and standard deviation and kurtosis in the chin-tuck position (*p*<0.05,*zval*<2.01,*ranksum*<81661). For skewness, nectar swallows showed a significant statistical difference in neutral position (*p*<0.02,*zval*<2.49,*ranksum*<83601), while in chin-tuck position water swallows has the lowest value (*p*<0.02,*zval*<2.45,*ranksum*<83338). The standard deviation was statistically different between water and nectar-thick (*p*=0.02,*zval*=1.41,*ranksum*=80110) as well as kurtosis between water and honey-thick (*p*=0.01,*zval*=2.95,*ranksum*=82742). Additionally, the entropy rate is observed to be significantly lower in water swallows than in the other two stimuli in both head position (*p*<<0.01,*zval*<−3.42,*ranksum*<71586). Water swallows showed a significantly higher value for the L-Z complexity in the chin-tuck position (*p*<0.05,*zval*<4.37,*ranksum*<86156), while a pairwise comparison between nectar-thick and honey-thick apple juices showed a difference for the entropy rate (*p*=0.02,*zval*=−1.71,*ranksum*=74989).

Also, we compared the extracted features between two accelerometer axes. Kurtosis in both head positions did not exhibit a significant statistical difference (*p*>0.05,*zval*<0.91,*ranksum*<78599). The standard deviation in the neutral head position and skewness in the chin-tuck position showed statistically significant differences between swallows for all stimuli (*p*<<0.01,*zval*<7.72,*ranksum*<92505). In the neutral position, skewness was significantly different between water and nectar-thick swallows (*p*<<0.01,*zval*<−4.64,*ranksum*<69137), while the standard deviation showed a significant difference for nectar-thick swallows in chin-tuck position (*p*<<0.01,*zval*<−3.07,*ranksum*<74432). The L-Z complexity and the entropy rate were also significantly different for all stimuli in both head positions (*p*<<0.01, *zval*<1.31,*ranksum*<715650).

### Frequency domain features results

Table 3 summarizes the values of the considered frequency features for swallowing sounds. The centroid frequency (*f*_*c*_) and the bandwidth (*BW*) were not affected by the fluid viscosity in the chin-tick position (*p*>0.05,*zval*<0.62,*ranksum*<79457), while the peak frequency (*f*_*p*_) had significantly higher values for water swallows in the chin-tuck position (*p*<0.04,*zval*<4.46,*ranksum*<82550). In the neutral head position, the peak frequency was significantly higher for water swallows than for honey-thick swallows (*p*=0.01,*zval*=2.29,*ranksum*<80912), while simultaneously the water swallows had significantly smaller bandwidth values than the honey-thick swallows (*p*=0.02,*zval*=2.49,*ranksum*=81282). The water swallows also had the smallest values for the centroid frequency in comparison to the other two types of swallows (*p*<<0.01,*zval*<3.81,*ranksum*<817740).

**Table 3:** Frequency domain features for swallowing sounds

		**Neutral position**			**Chin-tuck position**	
**Feature**	**Water**	**Nectar-thick**	**Honey-thick**	**Water**	**Nectar-thick**	**Honey-thick**
		**apple juice**	**apple juice**		**apple juice**	**apple juice**
*f*_*p*_	26.6 ±4.93	16.7 ±1.96	8.68 ±1.69	24.3 ±3.82	17.9 ±2.19	13.5 ±1.71
*f*_*c*_	446 ±45.4	464 ±51.6	493 ±65.7	739 ±66.1	802 ±69.5	767 ±73.4
*BW*	759 ±46.3	736 ±60.3	725 ±61.1	1161 ±68.5	1269 ±72.6	1236 ±71.8

The centroid frequency and bandwidth of the swallowing accelerometry signal in the A-P direction was not affected by fluid viscosity in the chin-tuck position (*p*>0.05,*zval*<0.93,*ranksum*<79973). However, in the A-P direction the centroid frequency and bandwidth has significantly higher value for water swallows in the neutral position (*p*<<0.01,*zval*<4.81,*ranksum*<86252). In the same direction, a pairwise comparison between water and honey-thick apple juice for the peak frequency showed differences in neutral position (*p*=0.006,*zval*=2.74,*ranksum*<823350), while in the chin-tuck position honey-thick swallows had statistically the lowest value (*p*<0.02,*zval*<4.33,*ranksum*<86078).

In the S-I direction, fluids did not impose any statistical differences on the centroid frequency in chin-tuck position, nor or the bandwidth in the neutral position (*p*>0.05,*zval*<1.11,*ranksum*<79528). The peak frequency was significantly different only between water and nectar-thick swallows in the head-neutral position (*p*=0.03,*zval*=2.06,*ranksum*=81347), and between water and honey-thick swallows in the chin-tuck position (*p*<0.01,*zval*=2.85,*ranksum*=83258). However, also in the S-I direction, the centroid frequency exhibited significant differences between water and honey-thick swallows in the neutral head position (*p*=0.01,*zval*=2.41,*ranksum*=81726), while water swallows had smaller bandwidth values than the nectar-thick swallows in the chin-tuck head position (*p*=0.02,*zval*=−2.2047,*ranksum*=73910). Table 4 summarizes frequency characteristics for swallowing accelerometry signals.

**Table 4:** Frequency domain features for swallowing accelerometery signals

		**Neutral position**			**Chin-tuck position**	
**Feature**	**Water**	**Nectar-thick**	**Honey-thick**	**Water**	**Nectar-thick**	**Honey-thick**
		**apple juice**	**apple juice**		**apple juice**	**apple juice**
*f*_*p*_ A-P	2.93 ±0.42	2.10 ±0.10	2.08 ±0.21	2.80 ±0.26	2.49 ±0.49	2.14 ±0.19
*f*_*p*_ S-I	6.09 ±0.44	5.57 ±0.48	5.12 ±0.29	5.83 ±0.46	5.72 ±0.49	5.28 ±0.62
*f*_*c*_ A-P	80.5 ±9.11	51.3 ±6.92	57.5 ±7.67	120 ±13.5	130 ±14.3	140 ±15.3
*f*_*c*_ S-I	63.2 ±8.33	59.5 ±10.4	62.4 ±10.1	105 ±11.6	110 ±10.7	108 ±8.89
*BW* A-P	141 ±14.1	100 ±9.78	112 ±12.2	215 ±15.7	244 ±17.9	243 ±17.6
*BW* S-I	94.8 ±9.89	89.7 ±9.23	85.8 ±11.1	174 ±13.3	225 ±15.9	218 ±15.8

While comparing statistical differences between the A-P and S-I directions, we found significant differences for the peak frequency for all stimuli in both head positions (*p*<<0.01,*zval*<−9.53,*ranksum*<598280). Furthermore, the centroid frequency is different for nectar-thick and honey-thick swallows in both head positions (*p*<0.03,*zval*<−2.16,*ranksum*<74720). The bandwidth was significantly different between the two directions for all stimuli (*p*<0.04,*zval*<5.29,*ranksum*<86842) in the neutral head position. Lastly, the bandwidth was significantly smaller for the S-I direction for water swallows in the chin-tuck position (*p*=0.02,*zval*=2.33,*ranksum*<82434).

### Time-frequency domain feature

**Figure 2:**
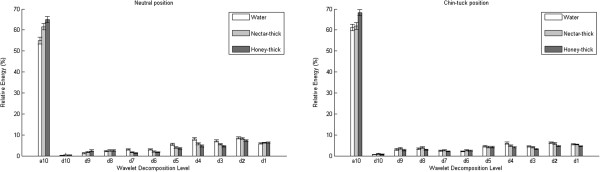
Mean relative energy per decomposition band for swallowing sounds.

**Figure 3:**
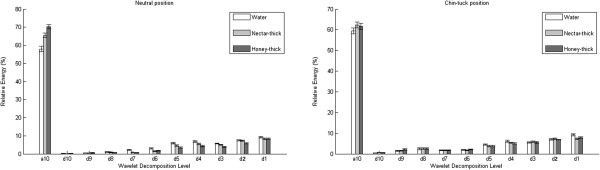
Mean relative energy per decomposition band for swallowing accelerometry signals in the A-P direction.

**Figure 4:**
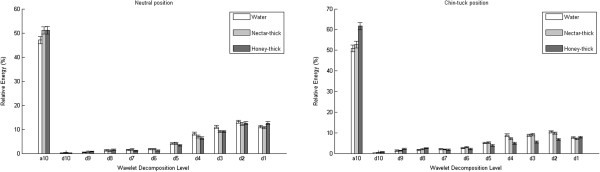
Mean relative energy per decomposition band for swallowing accelerometry signals in the S-I direction.

The relative energy decompositions are presented in Figures 2, 3, 4, while the wavelet entropy results for both swallowing sounds and accelerometry signals are summarized in Table 5.

**Table 5:** Wavelet entropies for swallowing sounds and accelerometry signals

		**Neutral position**			**Chin-tuck position**	
**Feature**	**Water**	**Nectar-thick**	**Honey-thick**	**Water**	**Nectar-thick**	**Honey-thick**
		**apple juice**	**apple juice**		**apple juice**	**apple juice**
WE	1.81 ±0.04	1.65 ±0.04	1.51 ±0.04	1.67 ±0.04	1.69 ±0.05	1.51 ±0.05
WE A-P	1.78 ±0.04	1.55 ±0.04	1.39 ±0.03	1.71 ±0.04	1.65 ±0.04	1.65 ±0.04
WE S-I	1.91 ±0.03	1.81 ±0.03	1.79 ±0.03	1.87 ±0.04	1.91 ±0.04	1.96 ±0.04

The wavelet analysis of the swallows showed that the viscosity of fluids had a major impact on the time-frequency structures of these signals. Let us first consider the swallowing sounds. From Figure 2, it is obvious that majority of the energy is concentrated on the first a10 level for both head maneuvers. Levels d10 and d9 in the neutral head position, and d8, d7, d6, d5, and d1 in the chin-tuck position were not affected by viscosity of the fluids (*p*>0.05,*zval*<1.77,*ranksum*<82367). In both head positions, water swallows had the statistically lowest value in the a10 level. However, water swallows had a higher energy concentration than the other two stimuli in the most of higher frequency levels (d8, d7, d6, d5, d4, d3,and d1 (*p*<0.04,*zval*<5.18,*ranksum*<86369)) in the neutral head position. Also, nectar swallows were statistically different from other stimuli for levels d4, d3 and d2 (*p*<0.03,*zval*<5.016,*ranksum*<86038). In chin-tuck head position, nectar swallows are shown to have statistical difference from other fluids in levels a10 and d3 (*p*<0.01,*zval*<3.11,*ranksum*<84170), while water swallow has the lowest value at level d10 (*p*<0.01,*zval*<−2.46,*ranksum*<73151). A pairwise comparison between water and honey-thick apple juice revealed significant differences for levels d4 and d2 (*p*<<0.01,*zval*<3.11,*ranksum*<83755), while water and nectar-thick apple juice were significantly different for the level d9 (*p*=0.01,*zval*=−2.41,*ranksum*=73515). Lastly, the wavelet entropy (*WE*) had a smaller value for the fluids with higher viscosity in the neutral head position (*p*<0.02,*zval*<5.14,*ranksum*<86278), while in the chin-tuck position, nectar swallows exhibited a significant difference from the other two swallows (*p*<0.02,*zval*<2.64,*ranksum*<83295).

Contrary to the previous study on swallowing accelerometry signals [44], a significant influence of fluid viscosity was noticed on the swallowing accelerometry signals from both directions. First, let us consider the relative energy decomposition of the swallowing accelerometry signals in the A-P direction. Similar to the swallowing sounds, most of the energy is concentrated in the a10 level for all fluids. Additionally, water swallows have the statistically lowest energy concentration in the a10 level (*p*<<0.01,*zval*<−1.17,*ranksum*<755616), which was not the case at higher frequencies, where water swallows had mostly higher energy concentration for both head maneuvers and both axes. The results for the A-P direction showed that the d10 and d9 levels in the neutral position and most of the levels in chin tuck position were not affected with viscosity of fluids. In the neutral head position, all stimuli showed a significant difference in the levels a10, d4 and d3, while water swallows exhibited higher energy concentrations in the d8, d7, d6, and d5 levels (*p*<0.01,*zval*<5062,*ranksum*<88134). Nectar-thick apple juice swallows revealed a significant difference in the d2 level (*p*<0.03,*zval*=0.41,*ranksum*=78187) for the neutral head position. In the chin-tuck head position, water swallows showed significant difference in level d1 (*p*<0.05,*zval*<5.62,*ranksum*<88134), while a pairwise comparison between water and nectar-thick showed a significant difference in the d10 level (*p*=0.01,*zval*=1.69,*ranksum*<74189). Lastly, in the A-P direction, the wavelet entropy had a significantly lower value for fluids with higher viscosity in the neutral position (*p*<0.02,*zval*<5.14,*ranksum*<86278). The wavelet entropy was not affected by viscosity in the chin-tuck position (*p*>0.05,*zval*<2.64,*ranksum*<83295).

In the S-I direction, levels d9, d6, and d2 in the neutral head position, and most of the levels in chin-tuck position did not show a significant statistical difference between stimuli. Water swallows were significantly different from other fluids in the a10, d10, d8, d7, d4 and d3 levels in the neutral position (*p*<0.04,*zval*<3.03,*ranksum*<84050), and in the d9 level in the chin-tuck position (*p*<<0.01,*zval*<−2.53,*ranksum*<73018). A pairwise comparison between water and honey-thick apple juice exhibited significant differences for the level d5 and d1 (*p*<0.01,*zval*<2.66,*ranksum*<82183) in the neutral position and for the level d10 (*p*=0.01,*zval*=−2.54,*ranksum*=72315) in the chin-tuck position. A pairwise comparison between water and honey-thick apple juice showed a significant difference in level d10 (*p*=0.01,*zval*=2.55,*ranksum*=72995) in chin-tuck head position, while pairwise between nectar-thick and honey-thick apple juice in level d1 (*p*<0.01,*zval*=−2.61,*ranksum*=73823) in neutral position showed difference. Also, the wavelet entropy had statistically the highest value for water swallows in the S-I direction (*p*<0.02,*zval*<2.73,*ranksum*<82639).

The relative energy distribution between the two axes were significantly different between each other. Levels a10, d10, d9, d8, d7, d5, d3 and d2 in the neutral position and levels a10, d4, d3 and d2 in the chin-tuck position showed difference between two axes for all three stimuli (*p*<<0.01,*zval*<8.91,*ranksum*<94965). Furthermore, swallows based on nectar-thick and honey-thick apple juices were also different between axes for the d1 level in the neutral head position and for the d5 level in the chin-tuck position (*p*<0.01,*zval*<−2.55,*ranksum*<72576). The relative energy distribution for water swallows was significantly different between two axes when considering the d5 level in the neutral position, and the levels d9, d8, d7 and d1 in the chin-tuck position (*p*<0.01,*zval*<3.74,*ranksum*<85110). However, the d4 level in the neutral head position and the levels d10 and d6 in the chin-tuck position were not significantly different between two axes (*p*>0.05,*zval*<0.33,*ranksum*<78820).

## Discussion

### Time domain features

Our results suggest that the time domain features for swallowing sounds are not different between nectar-thick and honey-thick fluids, while the water swallows had significantly different features from the other two fluids. These results imply that the difference in viscosity between nectar and honey have a limited effect on the extracted time domain features.

For the swallowing sounds, the negative value for skewness indicates that the probability distribution of amplitudes are mostly concentrated on the right side (i.e., stronger/louder amplitude values). Larger negative skewness values for swallows in chin-tuck position denote that swallows have larger (louder) amplitude values in the chin-tuck position than in the neutral position but viscosity did not affect amplitude. Also, kurtosis tends to be higher for higher viscosity fluids. Since kurtosis is a measure of “peakedness” of the amplitude probability distribution, the results imply that lower viscosity swallows would contain more variant amplitudes in the sound signal [26]. Clinically this result indicates that detection of varying viscosities of swallowed fluid might be possible with auscultation [23].

The entropy rate and the L-Z complexity for swallowing sounds were also influenced by viscosity of the fluid. According to Table 1, the mean value for the entropy rate is higher when viscosity increases, which implies that regularity of the signal is higher for more viscous fluids [37,38]. Similarly, a higher value for the L-Z complexity means that swallowing sounds are more complex and more unpredictable [45,46]. This is in agreement with previous studies of CA that have indicated large amounts of signal variability from subject to subject and swallow to swallow. From the Table 1, it is obvious that more viscous fluids have a lower mean value of the L-Z complexity, which implies that the signal complexity is lower for such fluids. The same results were provided by a previous study of the influence of viscosity on the accelerometer signal [44] where is implied that higher viscosity fluids tends to behave by better defined patterns. These findings indicate that further research into the specific characteristics of swallow sounds under various viscosity, posture, and other conditions, needs to be elucidated before auscultation will have more clinical value.

Swallowing accelerometry signals followed similar trends for the entropy rate and the L-Z complexity as shown in Table 2. These results confirm the findings from the previous study [44], which showed that regularity and predictability is higher for more viscous fluids. Also in the previous study, nectar-thick and honey-thick swallows had smaller negative values for skewness in the A-P direction. We confirmed the previous results for swallows in the chin-tuck position, but failed to confirm this trend for swallows in the neutral position.

### Frequency domain features

As shown in Table 3, thicker fluids yielded swallowing sounds with lower peak frequencies, which is already proven by a previous study about acoustic nature of normal swallows [47]. A similar trend was observed for swallowing accelerometry signals as well.

Comparing values for swallowing accelerometry signals from Table 4 with those values for swallowing sounds from Table 3, it can be concluded that swallowing sounds have much higher frequency content than the swallowing accelerometry signals. However, we observed similar trends for features extracted from these two types of signals. Bandwidth tends to be lower for higher viscosity fluids, which suggests that the more viscous fluids required more time for completion of the swallow [23]. The mean value of the centroid frequency for swallowing sounds is not dependent on viscosity, which implies that viscosity does not affect significantly spectral measure [48], which has also been observed for the accelerometer signal.

### Time-frequency domain features

The time-frequency decomposition of swallowing sounds showed that most of the signal energy is concentrated at lower frequencies, as was expected based on the frequency analysis of swallowing sounds. Thicker fluids have more energy on the first, lowest frequency level, since higher viscosity liquids produce a lower swallowing frequency [23]. Clinically, our results can be attributed to the increased total swallow duration, as previous studies showed that the oral and pharyngeal swallow durations increase when subjects swallow higher viscosity fluids [49,50]. We consider the wavelet entropy to describe spread of energy. According to Table 5, the mean value of wavelet entropy tends to be lower for higher viscosity fluids, because the energy concentration is higher at the first level for thicker fluids.

Similarly to the swallowing sounds, most of the energy from the accelerometry signals is concentrated at lowest frequency level (a10) for all stimuli. Also, the mean value of relative energy in a10 level tends to be higer for thicker fluids. These findings explain results for mean value of wavelet entropy which tends to be lower for thicker fluids.

### Remarks

According to results, more differences are observed for features in the neutral than in the chin-tuck head position for both swallowing sound and swallowing accelerometry signals. Furthermore, this study showed more statistical difference for a greater number of features extracted from swallowing accelerometry signals than the previous study [44]. In the previous study, most of the statistical differences were based on time domain features [44]. It should be mentioned that the previous study only considered data from 17 participants.

### Limitations and strengths of the present study

In this study, swallowing conditions have been administered to the subjects in a specific order (water, nectar-thick, honey-thick) implying that we cannot rule out the possibility that the order of presentation influenced the results. Also, no inference regarding swallowing physiology can be made from the results of this study as simultaneous imaging was not performed. Future research in this area could compensate for these limitations by including simultaneously acquired images and randomizing the order of presentation. However, this study has contributed to the general knowledge regarding the usefulness of CA as a screening method, as we need to clearly understand if there is any more value to CA than was previously reported. Future research should also focus on combining CA and swallowing accelerometry in a concurrent design (with imaging). The goal would be to determine if the detection accuracy of swallowing physiological impairments increases by combining these two sensors, or a higher accuracy is achieved by considering sensors independently. Also, such studies would enables us to understand the detection accuracy of specific physiologic events of these sensors compared to other screening methods.

## Conclusions

In this paper, we analyzed the effects of fluid viscosity on swallowing sounds in the normal and chin-tuck head positions. Swallowing sounds were collected from 56 healthy participants, and signal features were extracted from these sounds. Our analysis yielded several important conclusions. First, swallowing sounds contained lower frequency components than previously reported. Second, fluid viscosity greatly influenced some of the extracted features, especially in the frequency and time-frequency domains. Third, most of the time domain features exhibited differences between water and fluids with higher viscosity (i.e., nectar-thick and honey-thick fluids). The time domain differences were not dominant between nectar-thick and honey-thick fluids.

## Abbreviations

CA: Cervical auscultation; VFSS: Videofluoroscopic swallowing study; FIR: Finite impulse response; σ: Standard deviation; ν: Skewness; ϖ: Kurtosis; ρ: Entropy rate; L-Z: Lempel-Ziv complexity; fc: Centroid frequency; fp: Peak frequency; BW: Bandwidth.

## Competing interests

The authors declare that they have no competing interests.

## Authors’ contributions

IJ, JMD and BL carried out the data collection. IJ conducted all mathematical computations and statistical tests, and wrote the entire manuscript. JLC provided clinical insights regarding swallowing and critically revised the manuscript. ES designed the data collection protocol, suggested the main objective of the manuscript, provided supervision, and critically revised the manuscript. All authors read and approved the final manuscript.
